# Association Between Bisphenol A Exposure and Risk of All-Cause and Cause-Specific Mortality in US Adults

**DOI:** 10.1001/jamanetworkopen.2020.11620

**Published:** 2020-08-17

**Authors:** Wei Bao, Buyun Liu, Shuang Rong, Susie Y. Dai, Leonardo Trasande, Hans-Joachim Lehmler

**Affiliations:** 1 Department of Epidemiology, College of Public Health, University of Iowa, Iowa City; 2 Department of Nutrition and Food Hygiene, School of Public Health, Medical College, Wuhan University of Science and Technology Wuhan, Hubei, China; 3 State Hygienic Laboratory, University of Iowa, Iowa City; 4 Department of Pediatrics, New York University School of Medicine, New York; 5 Department of Occupational and Environmental Health, College of Public Health, University of Iowa, Iowa City

## Abstract

**Importance:**

Bisphenol A (BPA) is a major public health concern because of its high-volume industrial production, ubiquitous exposure to humans, and potential toxic effects on multiple organs and systems in humans. However, prospective studies regarding the association of BPA exposure with long-term health outcomes are sparse.

**Objective:**

To examine the association of BPA exposure with all-cause mortality and cause-specific mortality among adults in the United States.

**Design, Setting, and Participants:**

This nationally representative cohort study included 3883 adults aged 20 years or older who participated in the US National Health and Nutrition Examination Survey 2003-2008 and provided urine samples for BPA level measurements. Participants were linked to mortality data from survey date through December 31, 2015. Data analyses were conducted in July 2019.

**Exposures:**

Urinary BPA levels were quantified using online solid-phase extraction coupled to high-performance liquid chromatography–isotope dilution tandem mass spectrometry.

**Main Outcomes and Measures:**

Mortality from all causes, cardiovascular disease, and cancer.

**Results:**

This cohort study included 3883 adults aged 20 years or older (weighted mean [SE] age, 43.6 [0.3] years; 2032 women [weighted, 51.4%]). During 36 514 person-years of follow-up (median, 9.6 years; maximum, 13.1 years), 344 deaths occurred, including 71 deaths from cardiovascular disease and 75 deaths from cancer. Participants with higher urinary BPA levels were at higher risk for death. After adjustment for age, sex, race/ethnicity, socioeconomic status, dietary and lifestyle factors, body mass index, and urinary creatinine levels, the hazard ratio comparing the highest vs lowest tertile of urinary BPA levels was 1.49 (95% CI, 1.01-2.19) for all-cause mortality, 1.46 (95% CI, 0.67-3.15) for cardiovascular disease mortality, and 0.98 (95% CI, 0.40-2.39) for cancer mortality.

**Conclusions and Relevance:**

In this nationally representative cohort of US adults, higher BPA exposure was significantly associated with an increased risk of all-cause mortality. Further studies are needed to replicate these findings in other populations and determine the underlying mechanisms.

## Key Points

**Question:**

Is exposure to bisphenol A, a ubiquitous chemical of concern, associated with long-term risk of mortality?

**Findings:**

In a cohort study of 3883 adults in the United States, participants with higher urinary bisphenol A levels were at higher risk for death during approximately 10 years of observation. The adjusted hazard ratio comparing the highest vs lowest tertile of urinary bisphenol A levels was 49% higher for all-cause mortality and was 46% higher, albeit not statistically significant, for cardiovascular disease mortality.

**Meaning:**

The findings in this study suggest that a higher level of bisphenol A exposure is associated with an increased risk of long-term all-cause mortality.

## Teaser

This nationally representative cohort study assesses whether exposure to the ubiquitous chemical bisphenol A is associated with long-term risk of mortality from cardiovascular disease, cancer, or all causes among US adults.

## Introduction

Widespread exposure to bisphenol A (BPA) has emerged as a major public health concern.^1,2^ Bisphenol A is a high-volume industrial chemical produced primarily for the manufacturing of polycarbonate plastics and epoxy resins. Polycarbonate plastics based on BPA are used in many consumer products, such as plastic bottles, sports equipment, compact discs, some medical devices, and dental sealants and composites. Epoxy resins that contain BPA are used to line water pipes, coat the inside of food and beverage cans, and make thermal paper such as that used in sales receipts.^3,4^ As a result, BPA exposure to humans is ubiquitous from a variety of sources ranging from consumer products, food, and water to dust.^5^ National biomonitoring data in the United States show that BPA is detectable in more than 90% of urine samples in the general population.^6,7^ Currently in the United States, 12 states and Washington, DC have restrictions in place against BPA. In Europe, the European Chemical Agency has added BPA to the Candidate List of substances of very high concern.

Evidence from animal studies has shown that BPA has endocrine-disrupting effects.^8,9^ Exposure to BPA can disrupt endocrine function and metabolism, inducing the development of obesity and metabolic disorders.^10,11^ Exposure to BPA can also induce cardiac arrhythmias, accelerate atherosclerosis, decrease atrial contraction rate and force, and lead to cardiac remodeling in animal models.^12,13,14,15,16,17^ Moreover, previous epidemiologic studies have shown that BPA exposure is associated with an increased risk of obesity,^18,19,20,21^ diabetes,^22,23,24^ hypertension,^25^ and cardiovascular disease (CVD).^22,26^ However, most of the previous epidemiologic studies are cross-sectional, and prospective cohort studies examining the association of BPA exposure with long-term health outcomes are sparse. Although growing evidence suggests that BPA has potentially toxic effects on a variety of organs and systems in humans, the association between BPA exposure and risk of mortality remains unknown. In the present study, we used data from a nationally representative cohort to examine the association of BPA exposure with all-cause and cause-specific mortality in US adults.

## Methods

### Study Population

The National Health and Nutrition Examination Survey (NHANES) is a nationally representative health survey program of the civilian noninstitutionalized resident population in the United States. It is administered by the National Center for Health Statistics (NCHS) at the Centers for Disease Control and Prevention (CDC). The uniqueness of the NHANES program is that it not only collects questionnaire data through in-person interviews but also performs health examinations in the Mobile Examination Center and collects specimens for laboratory tests. The NHANES protocol has been approved by the NCHS Ethics Review Board. Written informed consent was obtained in NHANES from all participants. All participants received a cash payment for their time and effort and were compensated for transportation and baby or elder care during their participation.

For the present analysis, we included adults aged 20 years or older who participated in NHANES during the period from 2003 to 2008 and had available data on BPA measurements. We linked all participants to mortality data through 2015, which enabled approximately 10 years of observation for mortality outcomes. Individuals with CVD or cancer at baseline were excluded. The data analysis was performed in July 2019. The present study followed the Strengthening the Reporting of Observational Studies in Epidemiology (STROBE) reporting guideline for cohort studies.

### Assessment of BPA Exposure

Spot urine samples are collected in the NHANES program from participants aged 6 years or older. The BPA levels in urine samples were measured in one-third of randomly selected NHANES participants using online solid-phase extraction coupled to high-performance liquid chromatography–isotope dilution tandem mass spectrometry at the Division of Laboratory Sciences, National Center for Environmental Health, CDC. The lower limit of detection (LLOD) for BPA was 0.36 μg/L for the 2003 to 2004 samples and 0.40 μg/L for the 2005 to 2008 samples. For BPA levels below the LLOD (<7% of the samples provided by this study population), the NHANES staff assigned a value of the LLOD divided by the square root of 2. A detailed description of the methods of BPA measurement in NHANES was published previously.^6^

### Ascertainment of Mortality Outcomes

We used the NHANES Public-Use Linked Mortality File through December 31, 2015, which was linked by the NCHS to the National Death Index with a probabilistic matching algorithm to determine mortality status.^27^ The National Death Index is an NCHS centralized database of all deaths in the United States. Data about the underlying cause of death were used for case definition according to the *International Statistical Classification of Diseases, Tenth Revision*.^28^ Accordingly, the NCHS classified cardiovascular mortality as death from heart disease (codes I00-I09, I11, I13, and I20-I51) or cerebrovascular disease (codes I60-I69) and cancer mortality as death from malignant neoplasms (codes C00-C97). This approach has been previously validated by the CDC and used in many CDC reports.^29,30,31^

### Assessment of Covariates

Information on age, sex, race/ethnicity, educational level, family income, smoking status, alcohol drinking, physical activity, and dietary intake was collected using questionnaires. According to the 1997 US federal Office of Management and Budget standards, race/ethnicity was categorized into Hispanic (including Mexican and non-Mexican Hispanic), non-Hispanic White, non-Hispanic Black, and other. Family income was categorized as the ratio of family income to the federal poverty level (<1.0, 1.0-1.9, 2.0-3.9, and ≥4.0). A higher income to poverty ratio indicates a better family income status. Self-reported educational status was grouped as lower than high school, high school, and college or higher. In accordance with the NCHS classifications, individuals who smoked less than 100 cigarettes in their lifetime were defined as never smokers; those who had smoked more than 100 cigarettes but did not smoke at the time of survey were considered former smokers; and those who had smoked more than 100 cigarettes in their lifetime and smoked cigarettes at the time of survey were considered current smokers. Alcohol intake was categorized as none (0 g/d), moderate drinking (0.1 to 27.9 g/d for men and 0.1 to 13.9 g/d for women), and heavy drinking (≥28 g/d for men and ≥14 g/d for women). For physical activity, participants were asked an array of questions related to daily activities in the questionnaire, from which metabolic equivalent of task (MET) minutes per week was calculated. There have been some changes in physical activity questionnaires in NHANES since the 2007 to 2008 questionnaire. Therefore, physical activity for each participant was categorized according to standards appropriate for each cycle as follows: (1) below, less than 600 MET min/wk or 150 min/wk of moderate-intensity exercise; (2) meet, 600 to 1200 MET min/wk or 150 to 300 min/wk of moderate-intensity exercise; or (3) exceed, at least 1200 MET min/wk or 300 min/wk of moderate-intensity exercise. Dietary information was collected by 24-hour dietary recall interviews, from which total energy intake was calculated using the US Department of Agriculture Automated Multiple-Pass Method. We used the Healthy Eating Index-2010 (HEI-2010) to indicate the overall quality of diet (HEI-2010 scores range from 0 to 100, with 100 being the best-quality diet).^32^ Body weight and height were measured by trained health technicians following the NHANES Anthropometry Procedures Manual. Body mass index (BMI) was calculated as the weight in kilograms divided by the height in meters squared. Urinary creatinine level was measured using the Jaffé rate reaction, in which creatinine reacts with picrate in an alkaline solution to form a red creatinine-picrate complex.

### Statistical Analysis

The NHANES program uses a complex, multistage probability sampling design to represent a national, civilian, noninstitutionalized population in the United States. Therefore, sample weights, strata, and primary sampling units were applied following the NHANES Analytic Guidelines^33^ to account for the unequal probability of selection, oversampling of certain subpopulations, and nonresponse adjustment.

Mean values and proportions of baseline characteristics were compared using linear regression for continuous variables and logistic regression for categorical variables. We used Cox proportional hazards regression models to estimate hazard ratios (HRs) and 95% CIs for the associations between BPA exposure and risk of mortality. Follow-up time for each person was calculated as the difference between the NHANES examination date and the last known date alive or censored from the linked mortality file. In the fully adjusted model, we adjusted for age, sex, race/ethnicity, educational level, family income level, smoking status, alcohol intake, physical activity, total energy intake, overall diet quality indicated by HEI-2010 score, and BMI. To account for urine dilution, urinary creatinine levels were adjusted for in all the analysis models in this study, as previously recommended.^34^ Furthermore, we performed stratified analyses and interaction analyses to examine whether the association differed by age, sex, race/ethnicity, diet quality, physical activity, and obesity status. In addition, we conducted a sensitivity analysis using the E-value method^35,36^ to test whether and how our results were robust to potential unmeasured confounding. All statistical analyses were conducted using the survey modules of SAS software, version 9.4 (SAS Institute Inc). A 2-sided *P* < .05 was considered statistically significant.

## Results

We included 3883 adults aged 20 years or older (weighted mean [SE] age, 43.6 [0.3] years; 2032 women [weighted, 51.4%]) in this study. During 36 514 person-years of follow-up (median follow-up, 9.6 years; maximum follow-up, 13.1 years), 344 deaths occurred, including 71 deaths from CVD and 75 deaths from cancer. Participants with higher urinary BPA levels were more likely to be younger, male, and non-Hispanic Black and have lower educational level, lower family income, lower physical activity, higher total energy intake, poorer dietary quality, and higher BMI (Table 1).

**Table 1.** Characteristics of the Study Population, According to Tertiles of Urinary BPA Levels

Characteristic	Tertile of urinary BPA level, mean (SE)^a^			*P* value
	1	2	3	
No. of participants	1295	1301	1287	
Age, y	46.8 (0.5)	43.6 (0.5)	40.4 (0.4)	<.001
Sex, %				<.001
Male	42.5 (1.6)	51.1 (2.1)	52.1 (1.4)	
Female	57.5 (1.6)	48.9 (2.1)	47.9 (1.4)	
Race/ethnicity, %				<.001
Hispanic	12.0 (1.3)	14.3 (1.6)	13.9 (1.5)	
Non-Hispanic White	72.8 (2.4)	68.3 (2.5)	64.6 (2.4)	
Non-Hispanic Black	7.0 (1.1)	11.4 (1.4)	17.4 (1.9)	
Other	8.1 (1.4)	6.1 (0.9)	4.1 (0.8)	
Educational level, %				.04
<High school	15.9 (1.1)	17.7 (01.5)	18.8 (1.2)	
High school	23.4 (1.6)	24.5 (1.7)	28.0 (1.7)	
College or higher	60.7 (2.1)	57.8 (2.1)	53.2 (1.8)	
Family income to poverty ratio, %				<.001
<1.0	9.0 (0.7)	9.7 (1.0)	14.1 (1.4)	
1.0-1.9	17.0 (1.4)	19.9 (1.3)	20.3 (1.3)	
2.0-3.9	25.7 (1.3)	28.2 (1.4)	30.7 (1.6)	
≥4.0	43.7 (2.1)	36.6 (2.0)	29.7 (2.2)	
Missing	4.6 (0.6)	5.7 (0.8)	5.3 (0.8)	
Smoker, %				.02
Never	52.8 (1.5)	55.1 (1.6)	51.2 (1.9)	
Ever	23.6 (1.4)	22.3 (1.1)	20.2 (1.2)	
Current	23.6 (1.3)	22.6 (1.6)	28.5 (1.6)	
Alcohol drinking, %				.39
None	66.1 (1.7)	67.3 (1.6)	66.1 (1.9)	
Moderate	8.0 (0.8)	10.6 (1.0)	8.8 (1.1)	
Heavy	21.6 (1.5)	18.1 (1.2)	20.9 (1.5)	
Missing	4.3 (0.7)	4.1 (0.8)	4.2 (0.8)	
Physical activity category, %^b^				.02
Below	32.1 (1.4)	38.4 (1.5)	37.9 (1.6)	
Meet	16.6 (1.4)	14.1 (1.2)	13.2 (0.9)	
Exceed	51.2 (1.8)	47.5 (1.8)	48.9 (1.8)	
Total energy intake, kcal/d	2200.8 (34.6)	2287.0 (35.8)	2355.2 (0.5)	.001
HEI-2010 score	50.8 (0.6)	48.4 (0.6)	45.7 (0.5)	<.001
BMI category, %				
<25	41.1 (1.6)	29.7 (1.4)	33.2 (1.8)	<.001
25-29.9	31.0 (1.6)	33.1 (1.6)	29.5 (1.7)	
≥30	26.6 (1.2)	36.2 (1.8)	36.4 (1.9)	
Missing	1.3 (0.4)	1.1 (0.3)	0.9 (0.4)	

Abbreviations: BMI, body mass index (calculated as weight in kilograms divided by height in meters squared); BPA, bisphenol A; HEI-2010, Healthy Eating Index 2010; MET, metabolic equivalent of task.

^a^ Values are weighted mean (SE) for continuous variables and weighted percentages (SE) for categorical variables, except the number of participants.

^b^ Physical activity for each participant was categorized as follows: (1) below, less than 600 MET min/wk per week or 150 min/wk of moderate-intensity exercise; (2) meet, 600 to 1200 MET min/wk or 150 to 300 min/wk of moderate-intensity exercise; or (3) exceed, at least 1200 MET min/wk or 300 min/wk of moderate-intensity exercise.

Participants with higher urinary BPA levels were at higher risk of death during the follow-up (Table 2). After adjustment for age, sex, race/ethnicity, and urinary creatinine levels, participants with the highest tertile of urinary BPA levels had a 51% higher risk of all-cause mortality (HR, 1.51; 95% CI, 1.07-2.13) compared with those with the lowest tertile of urinary BPA levels. The association was not appreciably changed after further adjustment for other covariates. In the fully adjusted model including demographic characteristics, socioeconomic status, dietary and lifestyle factors, BMI, and urinary creatinine levels, the HR for all-cause mortality among participants with the highest tertile of urinary BPA levels compared with those with the lowest tertile was 1.49 (95% CI, 1.01-2.19). Similar results were observed for CVD mortality (HR, 1.46; 95% CI, 0.67-3.15), although this association was not statistically significant. Exposure to BPA was not associated with cancer mortality (HR, 0.98; 95% CI, 0.40-2.39). Stratified analyses showed that the observed associations of BPA exposure with mortality did not significantly differ by age, sex, race/ethnicity, diet quality, physical activity, or obesity status (Table 3; eTable in the Supplement). In the sensitivity analysis using the E-value to assess the potential of unmeasured confounding, the E-value was 2.34 for all-cause mortality for the point estimate and 1.11 for the lower confidence bound. The E-values for CVD mortality were 2.28 for the point estimate and 1.0 for the lower confidence bound; for cancer mortality, the E-value was 1.16 for the point estimate and 1.0 for the lower confidence bound.

**Table 2.** Association of Urinary BPA Levels With All-Cause Mortality and With Cause-Specific Mortality

Variable	Tertile of urinary BPA level, hazard ratio (95% CI)		
	1	2	3
Median BPA level, ng/mL	0.7	2.1	5.7
All-cause mortality			
Deaths per person-years	133 per 11897	100 per 12268	111 per 12349
Model 1^a^	1 [Reference]	1.06 (0.76-1.47)	1.51 (1.07-2.13)
Model 2^b^	1 [Reference]	1.10 (0.78-1.55)	1.49 (1.01-2.19)
CVD mortality			
Deaths per person-years	32 per 11897	18 per 12268	21 per 12349
Model 1^a^	1 [Reference]	1.13 (0.54-2.37)	1.68 (0.82-3.44)
Model 2^b^	1 [Reference]	1.10 (0.53-2.31)	1.46 (0.67-3.15)
Cancer mortality			
Deaths per person-years	31 per 11897	22 per 12268	22 per 12249
Model 1^a^	1 [Reference]	1.13 (0.45-2.81)	1.05 (0.44-2.47)
Model 2^b^	1 [Reference]	1.12 (0.48-2.63)	0.98 (0.40-2.39)

Abbreviations: BPA, bisphenol A; CVD, cardiovascular disease.

^a^ Adjusted for age, sex, race/ethnicity, and urinary creatinine levels.

^b^ Model 1 plus adjusted for educational level, family income status, smoking, alcohol drinking, physical activity, total energy intake, Healthy Eating Index 2010 score, and body mass index.

**Table 3.** Stratified Analyses for the Association of Urinary BPA Levels With All-Cause Mortality^a^

Variable	Tertile of urinary BPA level, hazard ratio (95% CI)			*P* value for interaction
	1	2	3	
Age, y				
<65	1 [Reference]	0.78 (0.46-1.31)	1.12 (0.65-1.93)	.24
≥65	1 [Reference]	1.04 (0.63-1.72)	1.64 (0.94-2.88)	
Sex				
Male	1 [Reference]	0.91 (0.57-1.45)	1.35 (0.82-2.22)	.28
Female	1 [Reference]	1.30 (0.81-2.09)	1.62 (0.94-2.81)	
Race/ethnicity				
White	1 [Reference]	1.23 (0.77-1.98)	1.90 (1.13-3.20)	.15
Non-White	1 [Reference]	0.72 (0.43-1.22)	0.87 (0.59-1.29)	
Diet quality^b^				
Lower	1 [Reference]	1.33 (0.90-1.98)	1.83 (1.11-3.02)	.24
Higher	1 [Reference]	0.80 (0.45-1.43)	1.15 (0.66-2.00)	
Physical activity^c^				
Lower	1 [Reference]	1.12 (0.70-1.81)	1.11 (0.71-1.75)	.90
Higher	1 [Reference]	0.96 (0.54-1.70)	1.78 (0.90-3.51)	
Obesity				
BMI <30	1 [Reference]	1.27 (0.84-1.91)	1.90 (1.27-2.84)	.25
BMI ≥30	1 [Reference]	0.72 (0.35-1.46)	0.98 (0.51-1.87)	

Abbreviations: BMI, body mass index (calculated as weight in kilograms divided by height in meters squared); BPA, bisphenol A.

^a^ Adjusted for age, sex, race/ethnicity, urinary creatinine levels, educational level, family income status, smoking, alcohol drinking, physical activity, total energy intake, Healthy Eating Index 2010 score, and body mass index.

^b^ Lower or higher diet quality was defined as a Healthy Eating Index 2010 score lower than the median score or the median score or above, respectively.

^c^ Lower or higher physical activity level was defined as below or meeting the physical activity guidelines, respectively.

## Discussion

In a prospective cohort of a US nationally representative sample, we found that BPA exposure was significantly and positively associated with all-cause mortality in adults. The association remained significant after adjustment for demographic characteristics, socioeconomic status, dietary and lifestyle factors, BMI, and urinary creatinine levels. There was a statistically nonsignificant association between BPA exposure and CVD mortality and no association between BPA exposure and cancer mortality.

To our knowledge, this is the first study examining the association of BPA exposure with risk of mortality. Our findings are in line with previous epidemiologic studies showing a significant association of BPA exposure with cardiometabolic disorders, including diabetes, hypertension, and CVD.^37,38,39^ In addition, BPA exposure is also associated with atherosclerosis,^40,41^ coronary artery stenosis,^42^ and reduction in heart rate variability in humans.^25^ The potential mechanisms underlying increased risk of mortality associated with BPA remain to be elucidated, which may include alteration in cardiac calcium handling, ion channel inhibition or activation, oxidative stress and inflammation, epigenetic modifications, and variations in transcriptome or proteome expression.^38,39^

Our findings may have major public health implications. Exposure to BPA is ubiquitous among humans, affecting more than 90% of the general US population.^6,7,43^ Although BPA exposure has decreased over time in the United States,^44^ it was still detected in 95.7% of urine samples from participants in NHANES during the period from 2013 to 2014.^7^ Given the wide range of potentially toxic effects of BPA in humans, it is imperative and important to minimize human exposure to BPA. Substitution of BPA with other bisphenol analogues, such as bisphenol F and bisphenol S, is becoming popular^7,45^; however, the health effects of those emerging BPA substitutes remain largely unknown.^20,46^ Evidence from animal and epidemiologic studies, although still limited, suggest that some BPA substitutes may have toxic effects similar to BPA.^45,47,48^

### Strengths and Limitations

This study has several strengths. We used nationally representative data from NHANES, which enables us to generalize our findings to a broader population. In addition, the abundant data from NHANES, including comprehensive information on demographic and socioeconomic characteristics, anthropometric measures, and diet and lifestyle factors, provide the opportunity to adjust for a variety of potential confounding factors. There are some limitations in this study. First, spot urine samples were used to measure BPA concentrations in NHANES because it is challenging and less feasible to collect 24-hour urine samples in a large sample size, nationally representative cohort. Although within-person and between-person variability exists, previous evidence shows that urinary concentrations of BPA derived from a single spot-sampling approach may adequately reflect the average exposure of a population to BPA when urine samples are collected from a sufficiently large population with random meal ingestion and bladder emptying times.^49^ Second, the NHANES Linked Mortality File identified causes of death through linkage to the National Death Index, which is based on death certificates. This approach has been previously validated by the CDC and used in many CDC reports^29,30,31^ and other relevant literature. However, we could not rule out the possibility of errors in the classification of the cause of death. Third, although many potential confounders were adjusted for, there might still be residual confounding by unmeasured factors. However, the sensitivity analysis using E-values showed that the association between BPA and all-cause mortality could only be negated by an unmeasured cofounder that had associations with both BPA exposure and all-cause mortality with an HR of at least 2.34. This HR was higher than the HRs of the known confounders that were measured in this study (range, 1.02-1.97). Therefore, it is unlikely that an unmeasured confounder would be more substantially associated with all-cause mortality than the known risk factors evaluated in the present study by having an HR exceeding 2.34.

## Conclusions

Our findings from a nationally representative cohort suggested that higher BPA exposure was significantly associated with an increased risk of all-cause mortality among US adults. The observed but statistically nonsignificant association between BPA exposure and CVD mortality warrants further investigation. In addition, further studies are needed to replicate our findings in other populations and determine the underlying mechanisms.

## Notes

Supplement. **eTable.** Stratified Analyses for the Association of Urinary BPA Levels With Cause-Specific Mortality
