# Supplementary material for: Association Between Bisphenol A Exposure and Risk of All-Cause and Cause-Specific Mortality in US Adults
Source: JAMA Netw Open. 2020 Aug 17;3(8):e2011620. doi: 10.1001/jamanetworkopen.2020.11620 (PMC7431989; doi:10.1001/jamanetworkopen.2020.11620)
Supplement: Supplement. — eTable. Stratified Analyses for the Association of Urinary BPA Levels With Cause-Specific Mortality [file jamanetwopen-3-e2011620-s001.pdf]

Supplementary Online Content

Bao W, Liu B, Rong S, Dai SY, Trasande L, Lehmler H-J. Association between bisphenol A exposure and risk of all-cause and cause-specific mortality in US adults. *JAMA Netw Open*. 2020;3(8):e2011620. doi:10.1001/jamanetworkopen.2020.11620

eTable. Stratified Analyses for the Association of Urinary BPA Levels With Cause-Specific Mortality

This supplementary material has been provided by the authors to give readers additional information about their work.

© 2020 Bao W et al. *JAMA Network Open*.

eTable. Stratified Analyses for the Association of Urinary BPA Levels With Cause-Specific Mortality

**Variable**		**Tertiles of urinary BPA levels**			**P for interaction**
		**Tertile 1**	**Tertile 2**	**Tertile 3**	
**CVD mortality**					
Age	<65 years	1 (reference)	0.70 (0.22-2.18)[ǂ]	1.67 (0.52-5.31)	0.71
	≥65 years	1 (reference)	1.17 (0.49-2.79)	1.71 (0.51-5.73)	
Sex	Male	1 (reference)	1.08 (0.35-3.29)	1.75 (0.50-6.19)	0.49
	Female	1 (reference)	0.98 (0.37-2.63)	1.11 (0.30-4.16)	
Race/ethnicity	Whites	1 (reference)	1.72 (0.71-4.16)	1.83 (0.51-6.49)	0.72
	Non-whites	1 (reference)	0.26 (0.07-1.00)	0.80 (0.35-1.82)	
Diet quality[#]	Lower	1 (reference)	1.45 (0.58-3.61)	0.91 (0.28-2.94)	0.46
	Higher	1 (reference)	0.64 (0.19-2.19)	2.27 (0.72-7.22)	
Physical activity[*]	Lower	1 (reference)	1.25 (0.55-2.84)	0.93 (0.30-2.90)	0.63
	Higher	1 (reference)	0.60 (0.13-2.71)	2.13 (0.40-11.36)	
Obesity	BMI < 30	1 (reference)	0.95 (0.32-2.80)	1.28 (0.50-3.29)	0.14
	BMI ≥ 30	1 (reference)	1.55 (0.43-5.66)	1.73 (0.29-10.2)	
**Cancer mortality**					
Age	<65 years	1 (reference)	1.03 (0.33-3.13)	1.08 (0.31-3.71)	0.29
	≥65 years	1 (reference)	1.00 (0.40-2.47)	1.03 (0.36-2.92)	
Sex	Male	1 (reference)	0.68 (0.29-1.59)	0.82 (0.36-1.87)	0.17
	Female	1 (reference)	1.75 (0.45-6.81)	1.28 (0.27-6.11)	
Race/ethnicity	Whites	1 (reference)	1.08 (0.30-3.81)	0.92 (0.27-3.10)	0.46

© 2020 Bao W et al. *JAMA Network Open*.

	Non-whites	1 (reference)	1.12 (0.45-2.81)	1.11 (0.48-2.60)	
Diet quality[#]	Lower	1 (reference)	1.64 (0.56-4.78)	1.24 (0.36-4.23)	0.21
	Higher	1 (reference)	0.40 (0.10-1.59)	0.60 (0.17-2.12)	
Physical activity[*]	Lower	1 (reference)	1.10 (0.28-4.26)	0.49 (0.10-2.37)	0.94
	Higher	1 (reference)	1.06 (0.40-2.81)	1.89 (0.74-4.80)	
Obesity	BMI < 30	1 (reference)	1.45 (0.57-3.68)	1.47 (0.59-3.64)	0.65
	BMI ≥ 30	1 (reference)	0.77 (0.17-3.59)	0.59 (0.14-2.44)	

[#] Lower or higher diet quality was defined as the healthy eating index (HEI) < the median score or ≥ the median score, respectively.

[*] Lower or higher physical activity level was defined as below or meeting the physical activity guidelines, respectively.

Adjustment for age, sex, race/ethnicity, urinary creatinine levels, education, family income status, smoking, alcohol drinking, physical activity, total energy intake, HEI2010 score, and BMI.

[ǂ] HR (95% CI).

© 2020 Bao W et al. *JAMA Network Open*.
